# Applying Meaning and Self-Determination Theory to the Development of a Web-Based mHealth Physical Activity Intervention: Proof-of-Concept Pilot Study

**DOI:** 10.2196/55722

**Published:** 2024-06-25

**Authors:** Stephanie A Hooker, A Lauren Crain, Jule Muegge, Rebecca C Rossom, Nicolaas P Pronk, Dhavan Prasad Pasumarthi, Gopikrishna Kunisetty, Kevin S Masters

**Affiliations:** 1 Division of Research and Evaluation HealthPartners Institute Minneapolis, MN United States; 2 Department of Psychology University of Colorado Denver Denver, CO United States

**Keywords:** physical activity, midlife, digital health, SMS text messaging, theory-based, meaning in life, mobile phone

## Abstract

**Background:**

Meaning in life is positively associated with health, well-being, and longevity, which may be partially explained by engagement in healthier behaviors, including physical activity (PA). However, promoting awareness of meaning is a behavior change strategy that has not been tested in previous PA interventions.

**Objective:**

This study aims to develop, refine, and pilot-test the Meaningful Activity Program (MAP; MAP to Health), a web-based mobile health PA intervention, theoretically grounded in meaning and self-determination theory, for insufficiently active middle-aged adults.

**Methods:**

Following an iterative user-testing and refinement phase, we used a single-arm double baseline proof-of-concept pilot trial design. Participants included 35 insufficiently active adults in midlife (aged 40-64 years) interested in increasing their PA. After a 4-week baseline period, participants engaged in MAP to Health for 8 weeks. MAP to Health used a web-based assessment and just-in-time SMS text messaging to individualize the intervention; promote meaning salience; support the basic psychological needs of autonomy, competence, and relatedness; and increase PA. Participants completed measures of the hypothesized mechanisms of behavior change, including meaning salience, needs satisfaction, and autonomous motivation at pretest (−4 weeks), baseline (0 weeks), midpoint (4 weeks), and posttest (8 weeks) time points, and wore accelerometers for the study duration. At the end of the intervention, participants completed a qualitative interview. Mixed models compared changes in behavioral mechanisms during the intervention to changes before the intervention. Framework matrix analyses were used to analyze qualitative data.

**Results:**

Participants were aged 50.8 (SD 8.2) years on average; predominantly female (27/35, 77%); and 20% (7/35) Asian, 9% (3/35) Black or African American, 66% (23/35) White, and 6% (2/35) other race. Most (32/35, 91%) used MAP to Health for ≥5 of 8 weeks. Participants rated the intervention as easy to use (mean 4.3, SD 0.8 [out of 5.0]) and useful (mean 4.3, SD 0.6). None of the hypothesized mechanisms changed significantly during the preintervention phase (Cohen *d* values <0.15). However, autonomy (*P*<.001; Cohen *d*=0.76), competence (*P*<.001; Cohen *d*=0.65), relatedness (*P*=.004; Cohen *d*=0.46), autonomous motivation (*P*<.001; Cohen *d*=0.37), and meaning salience (*P*<.001; Cohen *d*=0.40) increased significantly during the intervention. Comparison of slopes before the intervention versus during the intervention revealed that increases during the intervention were significantly greater for autonomy (*P*=.002), competence (*P*<.001), and meaning salience (*P*=.001); however, slopes were not significantly different for relatedness (*P*=.10) and autonomous motivation (*P*=.17). Qualitative themes offered suggestions for improvement.

**Conclusions:**

MAP to Health was acceptable to participants, feasible to deliver, and associated with increases in the target mechanisms of behavior change. This is the first intervention to use meaning as a behavior change strategy in a PA intervention. Future research will test the efficacy of the intervention in increasing PA compared to a control condition.

## Introduction

### Overview

Physical activity (PA) is one of the most beneficial behaviors for health and well-being [1,2]. The evidence is so overwhelming that some have argued that everyone could benefit from PA [3]. However, as many as 90% of American adults do not meet the recommended guidelines of ≥150 minutes of moderate-intensity PA per week (or ≥75 minutes of vigorous PA per week) [4,5].

Given the importance of engaging in regular PA for health, there has been considerable effort to develop interventions to increase PA, with modest results [6]. Interventions generally demonstrate short-term success but not long-term maintenance [7,8] and report varying attrition rates, with most participants dropping out in the first 6 months [9,10]. One potential explanation for the limited success in achieving maintenance is the lack of systematic, mechanistic approaches to PA intervention development. In particular, despite the abundance of research examining theoretical psychosocial influences on PA [11], theory is often poorly applied to behavioral interventions [12]. Consequently, new and innovative interventions focused on psychological mechanisms known to predict PA adoption and maintenance are essential to improve PA interventions.

### Theoretical Framework: Self-Determination Theory Integrated With Meaning

More than 80 health behavior theories have been identified in the scientific literature [13], and other researchers have attempted to understand cross-cutting behavior change strategies, linked with the mechanisms of change, to create comprehensive frameworks upon which interventions can be designed [14]. However, cross-theory frameworks still lack guidance on how to combine behavior change strategies in a meaningful way in interventions, whereas single behavior change theories can provide such guidance. Specifically, self-determination theory (SDT) [15] is a promising theory upon which to build innovative interventions to enhance long-term behavior change. SDT, and specifically the SDT subtheory called the process model of behavior change [16], posits that social environments and interventions that support the basic psychological needs of autonomy (feeling one’s behavior is self-organized and accompanied by a sense of volition), relatedness (feeling connected to others), and competence (feeling capable of achieving goals) foster the internalization of motivation and facilitate behavior change. SDT also posits that motivation exists on a continuum and that internally motivated behaviors are more likely to be maintained than behaviors that are externally motivated. Specifically, behavioral regulation ranges from amotivated (ie, not motivated to engage in the behavior at all) to intrinsically motivated (ie, motivated by enjoyment and inherent pleasure in the activity). Extrinsic motivation is divided into 4 behavioral regulation types, ranging from more externally motivated to more internally motivated: external regulation (ie, motivated by external rewards or punishments), introjected regulation (ie, motivated by the desire to avoid shame or guilt or to gain pride), identified regulation (ie, motivated because the behavior is consistent with a sense of identity), and integrated regulation (ie, motivated because the behavior is consistent with self-congruent values and goals). For simplicity, behavioral regulation is often bifurcated into controlled (external forms of regulation: external and introjected regulation) and autonomous (internal forms of regulation: identified, integrated, and intrinsic) [17].

Research demonstrates that individuals who report more internally regulated motivation (eg, motivated by congruence with the self or enjoyment) also engage in more PA and experience more positive psychological outcomes of exercise participation [18-22]. Previously inactive individuals participating in exercise interventions experience a decrease in external regulations and an increase in more internalized motivations over time [23], and more internalized motivations are associated with greater exercise persistence [24]. Four randomized controlled trials examining SDT-based interventions to increase PA [25], using motivational interviewing [26] frameworks, demonstrated that increasing self-determined motivation increased PA [27,28]. These interventions used 1:1 (eg, physician and patient), group, or email interventions to deliver content.

**Figure 1:**
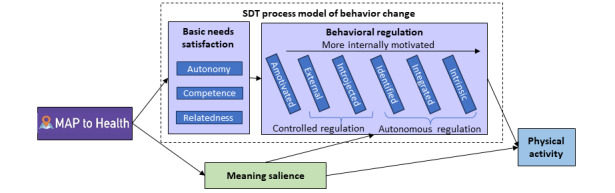
Conceptual model integrating self-determination theory (SDT) and meaning to increase physical activity. Mechanisms in the dashed box represent constructs from the SDT process model of behavior change. MAP: Meaningful Activity Program.

An implicit but somewhat overlooked aspect of SDT suggests that integrating or directly linking new behaviors with important and salient aspects of meaning in life increases the likelihood of long-term maintenance of the new behaviors [15,29,30]. Meaning in life is the sense that one’s life matters, makes sense, and has purpose [31]. Meaning and existential literatures explicate the basic human need to live a meaningful life. Research shows that people who engage in intrinsically meaningful life activities experience greater life satisfaction and well-being [32]. Although previous SDT intervention studies [25,33,34] assessed personal life goals and values, they did not deliberately integrate them with behavior change techniques or enhance awareness of meaning in life during the intervention. We hypothesize that meaning salience, or the extent to which individuals live with awareness of their sense of personal life meaning, is key to enhancing behavior change. Specifically, we hypothesize that an increased awareness of meaning salience supports self-regulatory strategies when engaging in behavior change and enhances internalized motivation to engage in the desired behavior [35]. Thus, our theoretical model (Figure 1) integrates meaning salience with the SDT process model of behavior change [16].

Individuals who live with awareness of a sense of meaning in life (ie, meaning salience) may be more motivated to engage in healthier behaviors [35,36]. Research supports this claim, and observational findings show that greater meaning is related to greater engagement in PA [37-41]. In previously inactive exercise initiates, on the days they experienced greater than average meaning salience, they engaged in more minutes of PA as well as more intense PA and were more likely to attend a fitness center [42]. Furthermore, in this same sample, a global sense of meaning in life was significantly and positively related to basic psychological needs satisfaction and internal motivation (key SDT mechanisms of change). Meaning in life, needs satisfaction, and internal motivation at baseline were significantly and positively related to PA 4 weeks later, suggesting that meaning may be another key behavioral determinant of PA [43]. We hypothesize that behaviors explicitly integrated within one’s life meaning are more likely to be maintained, particularly when the meaning salience is accentuated daily.

### Technology as a Mode of Delivery

Given that meaning salience is hypothesized to be a state that fluctuates within and across days, traditional face-to-face interventions pose challenges for enhancing meaning salience in the moment. Technological innovations, including mobile health (mHealth) and digital health interventions, offer innovative solutions to intervene during participants’ daily lives. SMS text messages and mHealth solutions can support a variety of different interventions, including delivering health education, offering treatment and prevention strategies, and increasing communication with patients and clinicians [44]. A recent meta-analysis showed that SMS text messages are an effective and flexible intervention strategy to increase PA [45]. SMS text messages can be standardized (same for all participants) or tailored (personalized to an individual) and delivered on all mobile phones, including low-cost devices. SMS text messages can also be used to deliver *ecological momentary interventions* (EMIs), or interventions delivered as people are going about their daily lives [46]. With this strategy, EMIs can encourage healthy behaviors by prompting or encouraging individuals to make a healthy choice. Evidence suggests that SMS text messaging and mHealth interventions have small to moderate positive effects on PA [45,47,48]. Digital health interventions that promote goal setting and planning, encourage self-monitoring, have prompts and cues, and are personalized are more effective than those that do not include these components [49]. However, meaning is a highly personal construct, and sending generic messages to increase meaning may not have the same impact as tailoring the message to an individual’s personal sense of meaning and reasons for wanting to be more active. Furthermore, when this personal motivation is combined with information about when participants are more likely to be active, they can be delivered *just in time* to encourage PA. To our knowledge, this is an approach that has not been attempted in prior mHealth PA interventions. Given this relatively new intervention approach, ensuring that this intervention delivery is acceptable to participants (ie, is easy to use and useful according to the technology acceptance model framework [50,51]) is an important step before larger-scale efficacy testing and implementation.

### Intervening During Midlife

Midlife (40-64 years of age) may be an important time to harness meaning and establish a healthy behavior pattern to improve health in later life. Evidence suggests that PA tends to decline as individuals age [52,53], although engaging in health behaviors during this time may be more important than ever to prevent the development of chronic disease and enhance healthy aging [54,55]. Furthermore, although midlife is a time when risk factors such as high blood pressure emerge [56], physical fitness in midlife is associated with delayed onset of chronic disease [57]. Importantly, midlife is also a time in human development when meaning becomes more salient [58]. Thus, adults in midlife may be a particularly apropos group in which to test the hypothesis that pairing meaning with PA enhances PA engagement.

### The Purpose of This Study

The goals of this study were to develop, refine, and pilot-test the Meaningful Activity Program (MAP; MAP to Health), a web-based mHealth intervention designed to enhance meaning salience and support SDT-based basic psychological needs of autonomy, competence, and relatedness to increase internalized motivation for PA. MAP to Health’s web-based assessment plus SMS text message modality (1) flexibly delivers EMIs to enhance meaning salience, (2) is resource sparing, and (3) can be scaled up to a large audience without requiring interventionists or continued software maintenance (as is required for smartphone apps). This study used the National Institutes of Health’s Science of Behavior Change and Behavioral Intervention Development and the Obesity-Related Behavioral Intervention Trials frameworks to develop the intervention and test its ability to modify the hypothesized mechanisms [59,60]. Specifically, we had three aims: (1) determine the intervention’s acceptability using the technology acceptance model framework [50,51], (2) examine the feasibility of delivering the intervention, and (3) determine whether the intervention was associated with the hypothesized mechanisms of change (meaning salience, basic needs satisfaction, and autonomous motivation). In exploratory analyses, we examined whether the intervention was associated with changes in well-being (life satisfaction, subjective vitality, and mood) and PA.

## Methods

### Study Design

This study used a single-arm double baseline pilot trial design. Participants completed a pretest assessment (−4 weeks) and then 4 weeks later completed a baseline assessment (0 weeks). They then started the intervention and completed midpoint (4 weeks) and posttest (8 weeks) assessments. After completing the posttest (8-9 weeks) assessments, participants completed a qualitative exit interview with a study coordinator. This study was preregistered on ClinicalTrials.gov (NCT05332145).

### Ethical Considerations

This study was approved by the HealthPartners Institute Institutional Review Board (A20-287). All 35 participants who began the study provided informed consent. Participants received up to US $250 for completing all self-report measures, sending back the accelerometers they wore for the study, and completing the exit interview.

### Participants

The eligibility criteria were chosen to identify a sample of insufficiently active midlife adults without serious medical or psychiatric conditions who were interested in increasing PA. Insufficiently active adults (individuals who engaged in 10-149 minutes of PA per week) [52] were chosen because they indicated interest in engaging in PA but had not yet made PA a regular habit. This group represents one-quarter of American adults [52] who could use support to meet PA guidelines. Participants were included if they were in midlife (40 to 64 years of age), were able to read and understand English, were insufficiently active, intended to increase PA in the next 30 days, had consistent access to a smartphone with text messaging capability, and were able to access the internet through a mobile phone or a computer. Individuals were excluded if they had a greater than minimal risk to starting a PA program (as indicated by a Physical Activity Readiness Questionnaire [61] score of >0); had a BMI of ≥40 kg/m^2^; were currently pregnant; had opted out of research; had a diagnosis of metastatic cancer, cardiovascular disease, serious psychiatric disorder (eg, bipolar disorder or schizophrenia), substance use disorder, or a cognitive or psychiatric condition that precludes the completion of questionnaires (including dementia); or had an Alcohol Use Disorders Identification Test–Consumption [62] score of >7.

### Recruitment

Potentially eligible participants were notified of the study in 1 of 3 ways: through email outreach to community groups and health and wellness champions, through a story on the health system intranet page about the study, or through mailed invitations to potentially eligible patients identified through electronic health record data pulls. In all communications, interested individuals clicked on a link to a self-screening form to determine eligibility. If eligible and still interested, they were asked to provide their contact information so that a member of the study team could reach out to them. Study team members telephoned participants, confirmed interest and eligibility, answered questions, emailed them study-related information (including an informed consent form), and scheduled them for an initial visit with the study coordinator.

### Intervention

#### Overview and Conceptual Framework

MAP to Health was a theory-driven web-based intervention integrated with REDCap (Research Electronic Data Capture; Vanderbilt University) data collection software [63] and Twilio text messaging software (Twilio Inc). The ultimate goal of the MAP to Health intervention was to help adults increase PA. Specifically, MAP to Health used theory (Figure 1) and mHealth technology to increase meaning salience and support basic psychological needs (supporting autonomy in PA activities, encouraging setting small goals to increase competence, and encouraging relatedness through promoting PA activities with important others) to, in turn, enhance internal motivation to engage in PA. By explicitly addressing meaning, the intervention overtly integrated PA with key life values to increase internal motivation for PA and PA maintenance.

#### Web-Based Assessment

The intervention was individualized by first having participants complete a web-based interactive assessment, using a motivational interviewing framework, to explore what was particularly meaningful to them and how PA was consistent with these goals. The intervention started with gathering information about past PA and reasons participants wanted to be more active. Next, the intervention provided a rationale for why it is important to connect PA to what is meaningful and valuable to them, followed by a short values clarification exercise in which participants connected PA to their top 2 life values. The intervention then highlighted the disconnect between participants’ values and current behavior and guided participants in setting small, achievable goals to increase their PA. Participants identified barriers to completing their goals and generated possible solutions to these barriers. Finally, they scheduled specific PA sessions for the following week by setting the activity, time, day, with whom, where, and what value was connected to it. After obtaining personalized information about meaning and PA goals, the web-based application generated personalized messages delivered as EMIs to patients via SMS text messaging.

#### SMS Text Messaging

For 8 weeks after the initial assessment, participants received SMS text messages on their personal mobile phones. The SMS text messages were delivered 15 minutes before the times that participants scheduled PA sessions and incorporated personalized messages about meaning (eg, for a person who derives meaning from work: “Good morning! Ready to go to the gym? Remember that being more active can help you take care of your health and be more efficient at work.”). These just-in-time messages encouraged awareness of meaning and PA to provide a counter stimulus to disrupt the daily stream of other stimuli that are barriers to PA. Thus, the goals of the messages were to (1) increase PA; (2) increase meaning salience; and (3) support basic psychological needs satisfaction to, in turn, internalize motivation to engage in PA.

#### Weekly Activity Scheduling

Each week, participants were prompted to log in to the web-based platform to update their PA schedule in the calendar for the coming week. Participants had the option of copying a schedule from a prior week and modifying it as they saw fit. Weekly scheduling ensured that participants continued to actively plan for PA and that SMS text messages were sent at appropriate times.

#### Intervention Development and Iterative Revision Process

The research team tested the functionality of the web-based assessment internally before conducting a rapid iterative testing and revision process with members of the target population. Four participants in each of 3 rounds completed the web-based assessment, rated the usability, the ease of use, and the theoretical fidelity of the intervention (refer to the *Measures* subsection for details), as well as the acceptability of individualized SMS text messages, and provided qualitative feedback on the intervention and suggestions for change. Participants offered several suggestions that were incorporated into the final intervention, including reducing the number of clicks, streamlining the assessment, adding short videos, and fixing technical glitches.

### Measures

#### Demographic Characteristics

Participants self-reported their age, sex, race, ethnicity, income, education, employment status, comfort with technology (ie, “How comfortable do you feel using technology?” rated on a scale ranging from 1=*not comfortable* to 4=*very comfortable*), and the use of wearable PA-tracking devices (ie, “Do you typically track your physical activity using a wearable device?” answered *yes* or *no*). PA history was measured using items developed by Marcus and Forsyth [64]. Specifically, participants reported how long it had been since they were regularly physically active.

#### Meaning Salience

The Meaning Awareness Scale (MAS) is a 6-item measure that assesses meaning salience [65]. The MAS is a revised version of the Thoughts of Meaning Scale [42]. Participants rated the extent to which they were aware of meaning over the past day (eg, “I was aware of the meaning in my life” rated on a 7-point Likert-type scale ranging from 1=*rarely* to 7=*very often*). Participants completed the MAS on 3 random days during each of the 4 assessment periods. Items were averaged each day for a total score, and total scores were averaged across these 3 days. Prior studies have found that the MAS is highly internally consistent and is positively associated with the presence of meaning in life, purpose in life, life satisfaction, positive mood, subjective vitality, daily spiritual experiences, and state mindfulness [65]. In this study, the internal consistency of the MAS was very high (Cronbach α=0.98) across the 4 time points.

#### Basic Psychological Needs Satisfaction

The Psychological Needs Satisfaction in Exercise Scale (PNES) [66] measured the satisfaction of the needs of autonomy (eg, “I feel free to exercise in my own way”), competence (eg, “I feel confident in my ability to perform exercises that personally challenge me”), and relatedness (eg, “I feel attached to my exercise companions because they accept me for who I am”) in exercise contexts. The PNES has 18 items that participants rate on a 6-point Likert-type scale ranging from 1=*false* to 6=*true*. Items were averaged within each subscale. In this study, the PNES demonstrated strong internal consistency reliability for all 3 subscales across the 4 time points (Cronbach α≥0.91).

#### Internal Motivation

Motivation internalization was measured using the Behavioral Regulations in Exercise Questionnaire, version 4 [67-69]. This 28-item questionnaire assesses motivations for exercise on the SDT continuum. There are 7 subscales with 4 items each: amotivation (eg, “I don’t see why I should have to exercise”), external regulation (eg, “I exercise because other people say I should”), introjected regulation, avoidance type (eg, “I feel guilty when I don’t exercise”), introjected regulation, approach type (eg, “I feel proud of myself when I persist”), identified regulation (eg, “It’s important to me to exercise regularly”), integrated regulation (eg, “I exercise because I value the benefits it gives me”), and intrinsic motivation (eg, “I exercise because it’s fun”). The subscales were combined using a bifurcation approach [17] and scored into 2 subscales: autonomous regulation and controlled regulation. Autonomous regulation was the average of the intrinsic, integrated, and identified scales, whereas controlled regulation was the average of the external and introjected scales. The autonomous regulation subscales and composite score demonstrated high internal consistency (Cronbach α≥0.79) across all time points. The controlled regulation subscales and composite score demonstrated inadequate and variable internal consistency reliability across the time points (composite score: Cronbach α=0.24-0.49); therefore, we chose not to include controlled regulation in the analysis.

#### Mood

Positive mood and negative mood were measured using a positive affect and negative affect scale previously used in a study of daily meaning and daily mood [32]. Eight items measured positive affect (relaxed, proud, excited, appreciative, enthusiastic, happy, satisfied, and curious), and 5 items measured negative affect (sluggish, afraid, sad, anxious, and angry). Participants rated their mood using a 5-point Likert-type scale ranging from 1=*very slightly or not at all* to 5=*extremely*. Participants completed the mood scale on 3 random days during each of the 4 assessment periods, and total scores were averaged across these 3 days. Both scales had acceptable internal consistency across the 4 time points (positive mood: Cronbach α=0.92-0.95 and negative mood: Cronbach α=0.74-0.91).

#### Subjective Vitality

The 6-item Subjective Vitality Scale (SVS) measures feeling active, alive, enthusiastic, and energetic (eg, “I feel alive and vital”) [70]. Participants rated the extent to which they generally felt this way on a 7-point Likert-type scale ranging from 1=*not at all true* to 7=*very true*. Items were summed for a total SVS score. The SVS demonstrated high internal consistency across all time points (Cronbach α≥0.89).

#### Life Satisfaction

The Satisfaction with Life Scale measures life satisfaction wherein participants rate their agreement with 5 statements rated on a 7-point Likert-type scale ranging from 1=*strongly disagree* to 7=*strongly agree* [71]. Responses were summed so that higher scores correspond with greater satisfaction with life. Internal consistency was very high across all 4 time points (Cronbach α=0.91).

#### PA Measurement

Participants wore ActiGraph wGT3X-BT accelerometers (ActiGraph, LLC) [72] for the duration of the study (12 weeks). Data were uploaded to the cloud using CentrePoint software (ActiGraph, LLC). Participants wore the accelerometer on their nondominant wrists during waking hours. The accelerometers measured total activity counts, minutes of moderate to vigorous PA (MVPA), and steps. A valid assessment week consisted of 4 monitored days (at least 1 weekend day and 3 weekdays), each with 10 hours of minimum wear time (determined by the best currently available wear-time algorithms) [73]. Data were analyzed in 60-second epochs; epochs with at least 2020 activity counts per minute were classified as MVPA and summed per week for each participant. Weekly summaries of PA (MVPA) and steps were averaged in each of three 4-week time periods for use in exploratory analyses: the 4 weeks before baseline assessment (weeks −4 to −1), the first 4 weeks of the intervention (weeks 1 to 4), and the second 4 weeks of the intervention (weeks 5 to 8).

#### Technology Acceptance

Participants answered 10 questions rating the extent to which they found the MAP to Health intervention easy to use (4 items) and useful (4 items) as well as their intentions to use the intervention in the future (2 items). Items were based on previous research [50,74] and modified for the purposes of this study. Participants rated each item on a scale ranging from 1=*strongly disagree* to 5=*strongly agree*. Items were averaged within each subscale and had high internal consistency (ease of use: Cronbach α=0.92, usefulness: Cronbach α=0.89, and intentions to use the intervention in the future: Cronbach α=0.98).

#### Intervention Fidelity to Theory

The Intervention Fidelity to Theory questionnaire is a 14-item questionnaire that assesses the extent to which the MAP to Health intervention adheres to the theoretical foundations of supporting autonomy, competence, and relatedness and prompts meaning salience [75]. Participants rated the extent to which they agreed with each item on a scale ranging from 1=*strongly disagree* to 5=*strongly agree*. Items were averaged within each subscale for a total score and had acceptable to high internal consistency (autonomy: Cronbach α=0.76, competence: Cronbach α=0.79, relatedness: Cronbach α=0.87, and meaning salience: Cronbach α=0.87).

### Procedure

Research team members confirmed participants’ eligibility and interest in the trial and scheduled them for a virtual meeting with the study coordinator. After registering and successfully logging in to the web portal, participants viewed and signed an electronic consent form. After consent was signed, REDCap triggered an email invitation to complete the first set of measures (pretest measures). In this same week, participants met with the study coordinator who explained the study, answered any questions, set up the ActiGraph accelerometer, and helped the participants set up the CentrePoint software on their mobile phones to periodically upload data. Participants were instructed to continue their typical activity levels until the intervention started (approximately 4 weeks later). Participants were automatically emailed assessments through REDCap at pretest (−4 weeks), baseline (0 weeks), intervention midpoint (4 weeks), and posttest (8 weeks) time points. During these same intervention weeks, participants received emails on 2 random days (2 weekdays and 1 weekend day) to report meaning salience and mood.

After the baseline assessments were complete, participants were invited to complete the web-based assessment to begin the MAP to Health intervention. SMS text messages were automatically sent 15 minutes before the times that participants had previously scheduled activities. Participants also received text messages 2 hours after a scheduled activity to report whether they had completed the activity. If yes, a congratulatory message was sent. If no, the message encouraged flexibility and problem-solving to find other times to be active. If participants did not have any scheduled activities for at least 48 hours, a message encouraging them to find times to be more physically active was sent. Participants were reminded to log in to the website at least weekly to schedule activities for the coming week.

At the end of the 8-week intervention, participants mailed back the ActiGraph and completed a semistructured exit interview with the study coordinator by telephone. The interview guide asked participants to describe (1) their impressions of the intervention, (2) what they liked about the intervention, (3) what they disliked about the intervention, (4) what they would change about the intervention, (5) how they felt about wearing the ActiGraph, (6) how useful it would be to have objective PA data integrated with the intervention web portal, and (7) their feedback on the research participation process (including compensation, surveys, and the clarity of instructions). The study coordinator typed the participant responses to each question during the interview; interview responses were exported into individual PDF files for analysis.

### Statistical Analysis

The goal of the analysis was to quantify the extent to which the intervention impacted the hypothesized theoretical mechanisms. As exploratory outcomes, we also assessed changes in well-being and PA. Participant ratings of meaning salience, basic psychological needs satisfaction, and autonomous regulation were compared across 4 time points: pretest (week –4), baseline (week 0), midpoint (week 4), and posttest (week 8). Cohen *d* values were calculated to provide standardized estimates of change at each time point compared to baseline. Linear mixed models assessed the significance and relative magnitude of these changes. The mixed models nested repeated mechanism measures within participants; estimated fixed effects for the pretest, midpoint, and posttest time points compared to baseline; and estimated a random participant intercept to account for within-person correlation across measures. A fixed effect linear contrast tested our hypothesis regarding the pattern of change in ratings over time; in particular, whether there was more change in the mechanisms from baseline to posttest time point than there had been from pretest to baseline time point. It estimated the relative change in each mechanism (Δ) by comparing the slope from the baseline-to-posttest period to the slope from the pretest-to-baseline period.

Without the intervention, we expected no change in hypothesized mechanisms; therefore, the preintervention time point parameter estimate would be close to 0. However, after intervention implementation, we hypothesized that participants would report increases in the theoretical mechanisms; therefore, the midpoint and posttest time point parameter estimates would be positive. Finally, it was expected that both planned contrasts would be positive and significant, meaning that the changes from baseline to the midpoint and posttest time points would be significantly more positive than the changes from pretest to baseline time points.

The same models that were used to examine changes in hypothesized mechanisms were used to examine exploratory well-being (life satisfaction and subjective vitality) outcomes. Comparably specified linear mixed models were used in exploratory analyses to assess accelerometer-documented PA (MVPA minutes per day and steps per day) across 3 time periods. The models nested repeated PA measures within participants, estimated fixed effects for the first 4 weeks and second 4 weeks of the intervention compared to before the intervention period, and estimated a random participant intercept.

Descriptive statistics were used to examine the ratings of technology acceptance model constructs and intervention fidelity to theory. Average ratings were considered sufficient if the mean reached the threshold of satisfactory agreement for each construct (ie, mean ≥4 on a 5-point Likert-type scale with 4=*agree*).

Qualitative exit interviews were analyzed using the framework method [76]. Two independent coders (SAH and JM) familiarized themselves with the interviews and generated a list of preliminary codes. Coding was conducted using NVivo 12 (Lumivero) [77]. After coding the first 5 interviews, the coders compared their coding, refined the codebook, and coded the remaining interviews. The coders compared codes for all interviews, and discrepancies were resolved through discussion. After coding was complete, NVivo 12 was used to generate a series of framework matrices for the team to identify overall themes. The 2 coders reviewed the text associated with each of the codes, in the context of the individual interview, and generated themes.

In this pilot, the aim was not to determine statistical significance but rather to assess whether MAP to Health elicited meaningful changes in the hypothesized mechanisms. On the basis of our previous observational study [43], the strengths of the observed relationships in Figure 1 were expected to be β=.55 (needs satisfaction to internal motivation), β=.30 (internal motivation to PA), β=.16 (needs satisfaction to meaning salience), and β=.10 (meaning salience to PA) to yield a small to moderate effect on PA. Therefore, if half of the intervention effect (Cohen *d* = 0.30 / 2 = 0.15) was mediated through the pathways in Figure 1, then the intervention would need to increase both needs satisfaction (estimated β=.50) and meaning salience (estimated β=.60) by Cohen *d*>0.55. The remaining Cohen *d* value (approximately 0.15) of the total effect would be represented as a direct effect on PA and could be obtained, at least in part, via unmeasured mechanisms. If the pretest versus baseline measures comparison yielded a difference of approximately 0, the midpoint versus baseline comparison would be similar in power to a paired *t* test. A 2-tailed paired *t* test with a sample size of n=35 was powered (0.80, α=.05) to detect differences of Cohen *d*≥0.49 in meaning salience and basic needs satisfaction. As such, the analyses may have sufficient power to detect meaningful within-person changes in the hypothesized mechanisms.

## Results

### Sample Characteristics

**Figure 2:**
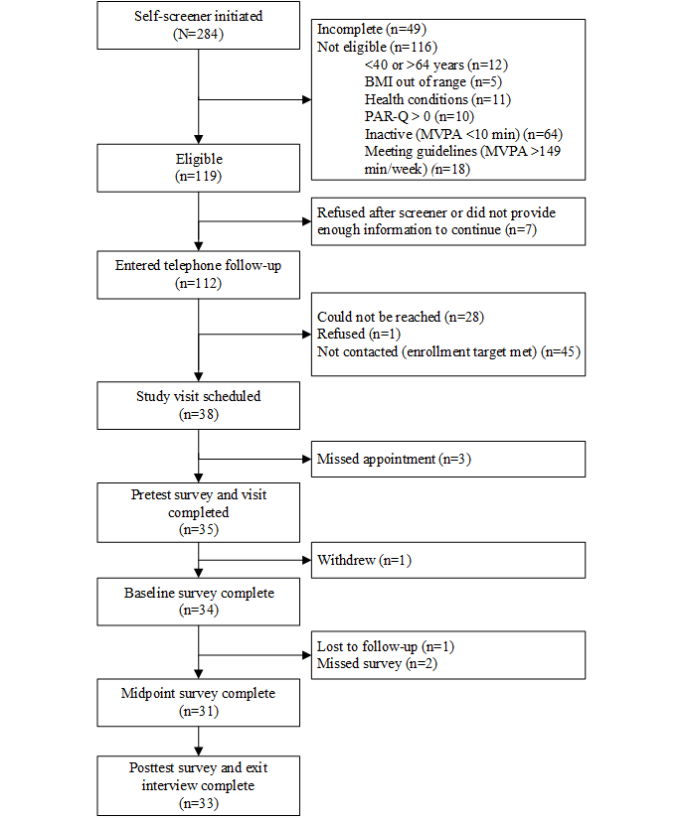
CONSORT (Consolidated Standards of Reporting Trials) flow diagram. MVPA: moderate to vigorous physical activity; PAR-Q: Physical Activity Readiness Questionnaire.

A total of 35 participants consented and began the study; 33 (94%) were retained through the posttest assessment (of the 2 participants who did not complete the study, 1 withdrew due to personal reasons, and 1 was lost to follow-up before starting the intervention; refer to Figure 2 for the CONSORT [Consolidated Standards of Reporting Trials] diagram). Of the remaining 33 participants, 2 (6%) missed the midpoint survey but completed the posttest survey. Participants were, on average, aged 50.8 (SD 8.2) years, predominantly female (27/35, 77%), highly educated (28/35, 80%), and had higher income (24/35, 69%; Table 1). At screening, participants reported that they engaged in an average of 57.6 (SD 37.9; range 10-130) minutes of MVPA per week.

**Table 1:** Participant characteristics (N=35).

Characteristic			Participants, n (%)
Sex (female)			27 (77)
**Race**			
	American Indian	1 (3)	
	Asian	7 (20)	
	Black or African American	3 (9)	
	White	23 (66)	
	Unknown	1 (3)	
**Ethnicity**			
	Hispanic or Latinx	2 (6)	
	Not Hispanic or Latinx	33 (94)	
**Employment status**			
	Employed full time	31 (89)	
	Employed part time	4 (11)	
**Education**			
	Some college	2 (6)	
	2-year degree	5 (14)	
	4-year degree	11 (31)	
	Postgraduate degree	17 (49)	
**Income (US $)**			
	40,000-59,000	1 (3)	
	60,000-79,000	4 (11)	
	80,000-99,000	6 (17)	
	≥100,000	24 (69)	
**Track physical activity (yes)**			17 (49)
	Apple Watch	12 (34)	
	Fitbit	4 (11)	
	Pedometer	1 (3)	
**Comfort using technology**			
	Moderately comfortable	13 (37)	
	Very comfortable	22 (63)	
**How do you access the internet?**			
	Smartphone	32 (91)	
	Computer	27 (77)	
	Tablet	11 (31)	
**How long since you last did regular PA^a^ (≥150 minutes of MVPA^b^ per week) (years)?**			
	<0.5	12 (34)	
	0.5-1	6 (17)	
	1-2	3 (9)	
	2-5	7 (20)	
	5-10	3 (9)	
	>10	1 (2)	
	Never	3 (9)	

^a^PA: physical activity.

^b^MVPA: moderate to vigorous physical activity.

### Acceptability, Intervention Fidelity to Theory, and Feasibility

At the posttest time point, participants rated the extent to which the technology was acceptable (useful, easy to use, and intentions to use the intervention in the future) and the intervention adhered to the theoretical principles of behavior change by supporting autonomy, competence, and relatedness as well as priming thoughts of meaning. For all measures, the target was ≥4.0 out of 5.0. Participants rated the intervention as useful (mean 4.3, SD 0.6) and easy to use (mean 4.3, SD 0.8), but the average rating for intentions to use the intervention in the future was slightly below the target (mean 3.8, SD 1.3). Participants rated the fidelity to theory highly for autonomy (mean 4.7, SD 0.5), competence (mean 4.5, SD 0.7), and meaning salience (mean 4.1, SD 0.8), but the average rating for relatedness was below the target (mean 3.7, SD 1.0).

Most of the participants (32/35, 91%) scheduled activities during at least 5 of the 8 weeks of the intervention, and 71% (25/35) scheduled at least 1 activity during all 8 weeks. Of the 35 participants, 2 (6%) never started the intervention, and 1 (3%) only scheduled activities for 1 week. On average, participants scheduled 51.9 (SD 39.7) activities over the 8 weeks, corresponding to an average of 6.5 (SD 5.0) activities per week. There was no evidence of declining participation over the 8 weeks, with the lowest number of participants scheduling an activity in the first week (28/35, 80%); in all other weeks (weeks 2-8), at least 1 activity was scheduled by 89% (31/35) or 91% (32/35) of the participants. Participants received an average of 6.2 (SD 4.7) preactivity SMS text messages per week; mean 1.3 (SD 0.7) SMS text messages per day; range 1-6 per person per day). Participants responded to an average of 80.4% (SD 17.7%) of the postactivity SMS text messages and confirmed that they completed 63.6% (SD 18.3%) of the scheduled activities. There was no evidence of a declining postactivity text message response rate over the 8 weeks, where the lowest response rates were seen during weeks 1 (mean 77.4%, SD 28.6%) and 4 (mean 76.1%, SD 29.4%), and the highest response rates were seen during weeks 2 (mean 85.2%, SD 21.5%), 5 (mean 85.3%, SD 18.9%), and 8 (mean 83.6%, SD 23.2%). Similarly, there was no evidence that reported PA completion rates declined over the 8 weeks, with the lowest completion rates in weeks 1 (mean 60.7%, SD 30.1%) and 4 (mean 56.2%, SD 31.8%) and the highest completion rates in weeks 2 (mean 69.7%, SD 26.1%), 5 (mean 74.4%, SD 24.1%), and 8 (mean 69.0%, SD 29.4%).

### Intervention Effects on Hypothesized Mechanisms

The primary analysis examined whether the intervention was significantly related to increases in the hypothesized mechanisms of change (basic needs satisfaction, autonomous regulation, and meaning salience) compared to change occurring before the intervention (Tables 2 and 3). For basic needs satisfaction, there were no significant changes on any of the 3 needs from before the intervention to baseline. Autonomy significantly increased from baseline to midpoint (*b*=0.37; *P*=.002; Cohen *d*=0.56) and from baseline to after the intervention (*b*=0.50; *P*<.001; Cohen *d*=0.76). Similarly, competence significantly increased from baseline to midpoint (*b*=0.45; *P*=.001; Cohen *d*=0.43) and from baseline to after the intervention (*b*=0.68; *P*<.001; Cohen *d*=0.65). For both autonomy and competence, the differences from baseline to after the intervention compared to those from before the intervention to baseline were significantly different from 0 and positive, suggesting that they increased more during the 8 weeks of the intervention than before starting the intervention (autonomy: Δ=0.61; *P*=.002; competence: Δ=0.85; *P*<.001). Relatedness did not significantly increase from baseline to midpoint (*b*=0.40; *P*=.10; Cohen *d*=0.27), but it did significantly increase from baseline to after the intervention (*b*=0.68; *P*=.004; Cohen *d*=0.46). The difference in relatedness slopes from baseline to after the intervention compared to that from before the intervention to baseline was in the expected direction but not different from 0 (Δ=0.65; *P*=.10).

Autonomous regulation increased, although not significantly, during the preintervention phase (*b*=0.73; *P*=.08; Cohen *d*=0.14) and increased significantly from baseline to midpoint (*b*=1.26; *P*=.004; Cohen *d*=0.25) and from baseline to after the intervention (*b*=1.70; *P*<.001; Cohen *d*=0.34). There was a trend suggesting that the change in autonomous regulation was greater during the intervention, but this difference was not significant (Δ=0.97; *P*=.17). An examination of the component regulations that make up autonomous regulation indicated that the largest changes were observed in identified regulation, whereas minimal to small changes were observed in intrinsic motivation and integrated regulation.

Meaning salience decreased slightly, although not significantly, during the preintervention period (*b*=−0.22; *P*=.09; Cohen *d*=−0.16), then increased from baseline to midpoint (*b*=0.25; *P*=.06; Cohen *d*=0.18), and significantly increased from baseline to after the intervention (*b*=0.55; *P*<.001; Cohen *d*=0.40). The difference in slopes from before the intervention and after the intervention compared to baseline was significant and positive (Δ=0.77; *P*=.001), indicating that meaning salience increased to a greater extent during the intervention.

**Table 2:** Changes in mechanisms and well-being over the course of the study.

			Mean (SD) over the course of the study					Mixed model, *P* value					Cohen *d*		
			Before the intervention, week −4	Baseline, week 0	Midpoint, week 4	After the intervention, week 8	Baseline vs before the intervention		Midpoint vs baseline	After the intervention vs baseline	(After the intervention vs baseline) vs (baseline vs before the intervention)	Before the intervention to baseline		Baseline to midpoint	Baseline to after the intervention
**Mechanisms**															
	**Needs satisfaction**														
		Autonomy	5.3 (0.6)	5.2 (0.7)	5.6 (0.6)	5.7 (0.6)	.33		.002	<.001	.002	−0.170		0.560	0.760
		Competence	4.5 (1.0)	4.3 (1.3)	4.7 (0.9)	5.0 (0.9)	.18		.001	<.001	<.001	−0.170		0.430	0.650
		Relatedness	3.6 (1.4)	3.7 (1.5)	4.0 (1.4)	4.3 (1.6)	.91		.10	.004	.10	0.020		0.270	0.460
	**Behavioral regulation**														
		Intrinsic motivation	17.6 (6.1)	18.6 (7.1)	18.9 (5.7)	19.6 (7,4)	.13		.14	.05	.78	0.140		0.140	0.180
		Integrated regulation	15.3 (7.1)	16.5 (7.5)	17.5 (7.3)	18.2 (7.9)	.10		.01	.004	.45	0.130		0.212	0.230
		Identified regulation	23.9 (4.2)	24.3 (4.4)	25.4 (2.6)	26.4 (2.4)	.50		.01	<.001	.03	0.090		0.340	0.590
		Autonomous regulation	18.9 (5.0)	19.8 (5.5)	20.6 (4.5)	21.4 (5.1)	.08		.004	<.001	.17	0.140		0.250	0.340
		Meaning salience	5.3 (1.4)	5.1 (1.5)	5.3 (1.4)	5.6 (1.2)	.09		.06	<.001	.001	−0.160		0.180	0.400
**Well-being**															
	Life satisfaction		25.4 (5.6)	25.2 (6.1)	26.8 (5.8)	27.4 (6.2)	.56		.007	<.001	.007	−0.050		0.260	0.380
	Subjective vitality		27.4 (7.3)	27.9 (7.9)	28.4 (7.6)	30.4 (7.6)	.60		.11	<.001	.07	0.050		0.170	0.370
	Positive affect		3.4 (0.7)	3.2 (0.8)	3.5 (0.8)	3.5 (0.8)	.13		.02	.007	.02	−0.220		0.350	0.400
	Negative affect		1.8 (0.7)	1.8 (0.5)	1.6 (0.4)	1.6 (0.5)	.33		.02	.003	.02	0.160		−0.410	−0.520

**Table 3:** Changes in physical activity (PA) over the course of the study.

PA	Mean (SD) over the course of the study			Mixed model, *P* value			Cohen *d*	
	Before the intervention (weeks −4 to −1)	First 4 weeks of the intervention (weeks 1 to 4)	Second 4 weeks of the intervention (weeks 5 to 8)	First 4 weeks of the intervention vs before the intervention	Second 4 weeks of the intervention vs before the intervention	Before the intervention to first 4 weeks of the intervention		Before the intervention to second 4 weeks of the intervention
Wear, minutes per day	899.3 (130.2)	898.3 (146.8)	893.6 (151.2)	.86	.81	−0.02		−0.03
MVPA^a^, minutes per day	142.5 (56.4)	141.7 (55.5)	144.7 (49.6)	.67	.98	0.03		0.00
Steps per day	4113 (2108)	4233 (2006)	4555 (1949)	.64	.17	0.04		0.13

^a^MVPA: moderate to vigorous physical activity.

### Intervention Effects on Well-Being

The same analytic approach was used to examine the preliminary impacts of the intervention on the measures of well-being. None of the well-being measures significantly changed during the preintervention period (life satisfaction: *b*=−0.31; *P*=.56; Cohen *d*=−0.05; subjective vitality: *b*=0.40; *P*=.60; Cohen *d*=0.05; positive affect: *b*=−0.17; *P*=.13; *d*=−0.22; and negative affect: *b*=0.09; *P*=.33; Cohen *d*=0.16). Except for subjective vitality, all measures of well-being demonstrated significant and small improvements from baseline to midpoint (life satisfaction: *b*=1.53; *P*=.007; Cohen *d*=0.26; subjective vitality: *b*=1.25; *P*=.11; Cohen *d*=0.17; positive affect: *b*=0.27; *P*=.02; Cohen *d*=.35; and negative affect: *b*=−0.22; *P*=.02; Cohen *d*=−0.41), and well-being on all measures (including subjective vitality) improved from baseline to after the intervention (life satisfaction: *b*=2.23; *P*<.001; Cohen *d*=0.38; subjective vitality: *b*=2.84; *P*<.001; Cohen *d*=0.37; positive affect: *b*=0.31; *P*=.007; Cohen *d*=0.40; and negative affect: *b*=−0.28; *P*=.003; Cohen *d*=−0.52). The differences in slopes were all in the expected directions, indicating that well-being improved to a greater extent during the intervention than before the intervention (life satisfaction: Δ=2.54; *P*=.007; subjective vitality: Δ=2.44; *P*=.07; positive affect: Δ=0.48; *P*=.02; and negative affect: Δ=−0.37; *P*=.02).

### Intervention Effects on PA

The average weekly minutes of MVPA in three 4-week periods (before the intervention to baseline, baseline to midpoint, and midpoint to after the intervention) were assessed as an exploratory outcome. Across the 12-week study period, accelerometer-documented MVPA remained high and stable (mean 142.9, SD 53.4 minutes per day), particularly in comparison to the self-reported average of 57.6 (SD 37.9) minutes of MVPA per week at study enrollment. Documented MVPA did not change significantly from the 4 weeks before baseline through the first 4 weeks of the intervention (*b*=1.62; *P*=.67; Cohen *d*=0.03) or from the 4 weeks before baseline through the second 4 weeks of the intervention (*b*=0.12; *P*=.98; Cohen *d*=0.00). By contrast, steps per day were in the low average range across the study (mean 4291, SD 2010). Steps per day did not change from the 4 weeks of the preintervention period to the first 4 weeks of the intervention (*b*=86.6; *P*=.64; Cohen *d*=.04) or to the second 4 weeks of the intervention (*b*=266.8; *P*=.17; Cohen *d*=.13).

### Qualitative Feedback

Most of the participants (33/35, 94%) participated in the qualitative exit interview. Themes and supportive quotes are presented in Table 4. Participants liked many aspects of the intervention, including setting goals, scheduling activities, and receiving text messages; reflecting on values, behaviors, and motivations; and the autonomy and flexibility of being able to choose their goals and activities. Participants disliked aspects of the research design, including the accelerometer; the double baseline pretest design (having to wait 4 weeks to start the intervention); and for participants who preferred to exercise alone, having to answer questions about relatedness or receive messages encouraging exercising with others. Participants recommended several changes to the technology to enhance the ease of use and usability, including changing the scheduler to be more flexible (allowing scheduling of same-day activities or recording of past activities); adding a way to visualize progress toward goals; including a way to allow participants to adjust or reevaluate their goals, values, or barriers; and allowing patients to continue receiving text messages after 8 weeks.

**Table 4:** Themes and supportive quotes of participants’ intervention experience from the qualitative exit interview.

Theme				Supportive quotes
**Theme 1**				
	Participants liked many aspects of the program and perceived that it helped them integrate PA^a^ into their lives.		“This was an innovative program that engaged me, gave me strategies, and planned my time and got me hooked for good.” [ID 201326]	
	**Subtheme 1a**			
		Scheduling and text reminders helped participants plan, find opportunities for PA, and follow through with their goals.	“I really enjoyed getting the messages. It helped me make the habit. Prior to this I wasn’t doing much. I used to sit all day long. After starting this program, I had less pain and was more flexible. My muscles used to be tight and not moving. This was a commitment to my health. I can go outside and go for a walk. Not just because I have to exercise, but because my health and well-being was important.” [ID 121424] “Frequent reminders kept me motivated. It definitely increased my activity. At times when I definitely didn’t feel like going out, like when it snowed out. Even in weeks when I scheduled 3-4 activities, I did 5.” [ID 121293]	
	**Subtheme 1b**			
		Participants enjoyed setting goals and reflecting on values, behaviors, and motivations.	“I really need to be intentional and really look at the values and this program really helped me to think. Physical activity is really good for me, but I just get busy. Setting those values really helped me, pushed me. It made me be intentional to go out with my family.” [ID 201319] “I also thought that it was really interesting to think about activity through the lens of values. One of my values is travel and we are going on a trip later this year and I just had never thought about how those are connected.” [ID 121492]	
	**Subtheme 1c**			
		Participants liked the autonomy that came with the program.	“The flexibility. It wasn’t telling me to do certain things at specific times, it allowed me to pick and choose. And if I didn’t do it, it said that’s okay. I’ve failed at other programs that enforce so much that I couldn’t accommodate in my busy life.” [ID 201314]	
**Theme 2**				
	Participants did not like some aspects of the research design, including having to wait to start the intervention, wear the accelerometer, or be prompted to be active with others.		“I was so excited to participate, and I had been trying to be more active, but then I had to be normal. I understood that was part of the study, but it was demotivating, and I lost momentum. The timing was hard because it was after the new year.” [ID 121334]	
	**Subtheme 2a**			
		Most participants did not like the PA monitoring device and suggested switching to commercially available devices that emphasize comfort.	“The device. It could be smaller. I got lots of comments at the gym. It would be great if you could integrate it into the Apple Watch. I would like something smaller that didn’t stick out too far. I wasn’t hoping for the end, but I was ready to be done wearing the device.” [ID 121367]	
	**Subtheme 2b**			
		For participants who mainly exercised alone, relatedness questions that asked about exercising with others and texts that encouraged being active with others felt like they didn’t apply.	“I don’t like to work out with other people, I like to work out on my own. It’s space that I create for myself. And all the texts were so focused on doing things with other people so that added to the frustration. It didn't shift to me and my preference. It kept gnawing at me that I wasn’t going to work out with other people.” [ID 121344]	
**Theme 3**				
	Changes to the intervention could make it more user friendly.		“I started to think ahead and look at my schedule and think about what I wanted to do. But there would be times when I wanted to do a group class and I would switch to do something different and there was no way to record that.” [ID 121389]	
	**Subtheme 3a**			
		Have more flexibility in scheduling same-day activities or recording past activities.	“I didn’t like that you couldn’t change your activities the day of. You plan out your week and then the day of you do something else and there was no way to change that in the planner. I don’t like predicting a week ahead of time what I’m going to do. I want to plan the day of. Half the time I ended up doing other things and there was no way to change the activities.” [ID 10014]	
	**Subtheme 3b**			
		Have a way to visualize progress, such as monitoring steps, heart rate, and activity level and meeting goals, that would support accountability toward goals and an opportunity to readjust expectations.	“It would be nice to see a graph or a dashboard to see how you improve. I think that would help to maintain and understand the dynamic of what you are doing. It would be nice to see steps, distance walked, by week or per day. I don’t do high-intensity exercise, so not sure about heart rate, but maybe it would help to see the different exercises.” [ID 121424]	
	**Subtheme 3c**			
		Include a way to allow participants to adjust their goals, values, motivations, and barriers over time.	“It would be good to have 1 or 2 opportunities to change up those goals. After getting the same one repeatedly, maybe there’s a different message I want to give myself.” [ID 10022] “I think I had to guess about what the barriers were, but you don’t really know until you get into it.” [ID 121462]	
	**Subtheme 3d**			
		Although most participants thought the program length was just about right, many would have liked to continue receiving text messages and others wanted to be done wearing the PA monitor.	“Honestly, I could have done it longer if it weren’t for the accelerometer.” [ID 121328] I would have loved to have had it for 12 weeks instead of 8. It had a positive effect for goals for health. I signed up for a gym membership, I bought a wristwatch that will track activity.” [ID 201326]	

^a^PA: physical activity.

## Discussion

### Principal Findings

This proof-of-concept pilot trial of a novel theory- and technology-driven intervention (MAP to Health) demonstrated that the intervention was used and well accepted by participants and was associated with changes in the hypothesized mechanisms. Specifically, participants rated the intervention as easy to use and useful and agreed that it supported their basic needs of autonomy, competence, and relatedness and promoted meaning salience. In qualitative interviews, participants noted that they liked setting goals, scheduling activities, and receiving reminder texts, as well as the opportunities to reflect on values, behaviors, and goals. Participants disliked the accelerometer and double baseline design and suggested changes to the scheduler and web interface to make it more user friendly. To our knowledge, MAP to Health is the first intervention that targeted meaning salience as a mechanism to increase PA.

Using a web-based assessment, MAP to Health delivered personalized text messages just in time to encourage PA. These messages primed participants to think about what is meaningful to them and the deeper reason for wanting to increase PA, while supporting the SDT-based needs of autonomy, competence, and relatedness. As hypothesized, the intervention was associated with moderate to large increases in needs satisfaction and meaning salience and small to moderate increases in internalized motivation. Previous studies have demonstrated that these mechanisms are related to PA in previously inactive people trying to increase PA [23,24,42,43]; thus, they are viable targets for increasing PA. This study demonstrates that the MAP to Health intervention can use personalized EMIs delivered through text messaging to sufficiently engage these targets.

Another goal of this study was to assess the acceptability and feasibility of using the MAP to Health intervention in midlife adults. Participants were highly engaged in the digital intervention, with 91% (32/35) of the participants scheduling activities during at least 5 out of 8 weeks and 71% (25/35) scheduling activities during all 8 weeks. Engagement and optimal use in digital interventions can be somewhat difficult to define, and many digital interventions suffer from low use [78,79]. Thus, it was highly possible that participants would complete the initial assessment but never log back in to set new goals and schedule activities. However, the intervention proved to be sufficiently engaging to keep participants coming back to the tool without a human coach encouraging use.

As an exploratory outcome, we measured PA to discern whether there was a signal that the intervention was associated with changes in PA. However, we found that the number of accelerometer-documented minutes of MVPA was consistently very high across the preintervention and postintervention periods (nearly meeting PA weekly guidelines each day), an unlikely finding given that the intervention recruited insufficiently active adults. To further understand this, we examined step counts which were in the low average range and consistent across the 2 periods. These data could be interpreted in several ways. First, it could be that these participants, all of whom reported being insufficiently active at screening (average of 58 minutes of MVPA per week), became very active when starting the study, even before being exposed to the intervention. We did hear from some participants that they were very excited about joining the study and did not like having to wait to start the intervention; therefore, it is possible that they started being very active at study enrollment. However, this interpretation seems unlikely because motivating the average participant to go from insufficiently active to highly active would likely take more intervention than simply signing up for a research study. The step measure was also not consistent with the number of MVPA minutes that were generated by the accelerometer, additionally suggesting that the accelerometer overestimated MVPA. There are reports that wrist-worn accelerometers tend to overestimate energy expenditure [72,80], and although we tested whether applying a correction would solve the problem, this only increased the estimates of MVPA. Thus, our interpretation is that our measurement had noteworthy error variance, and we cannot understand the extent to which the intervention increased (or did not increase) PA. In addition, most of the participants did not like the accelerometer, which suggests that a different measurement tool is needed for future studies. Specifically, using accelerometers that are designed to maximize comfort and usability, such as commercially available devices, may be helpful in increasing participant acceptability.

Qualitative exit interviews provided rich data to understand participants’ experiences in the intervention and offered several potential changes for future iterations. One reason participants did not like the accelerometer (in addition to the lack of comfort) was that it did not provide a way to monitor their PA. Participants desired some way to visualize their PA data and their progress, and many suggested integrating the web-based application with a commercial PA-tracking device. Although the accelerometer was meant as a PA measure for the research study, participants viewed it as part of the intervention, which influenced their view of the intervention itself. However, adding a way to visualize activity data and goal progress would enhance participants’ abilities to self-monitor and adjust their behavior and their goals [81]. This would be a positive enhancement of the MAP to Health intervention that would likely improve its impact. Participants also requested the ability to schedule activities on the same day and record past activities, as well as reassess goals, values, and barriers, to further personalize the intervention to their needs because these variables may change over time. Creating a flexible digital tool such as this could better support their basic needs of autonomy and competence.

Targeting relatedness as a mechanism in an individually delivered digital intervention proved challenging; it was 1 area that was below the target for fidelity to theory, and it increased to a lesser extent than the satisfaction of other needs. Prior SDT interventions have used group formats to foster relatedness among participants [82,83]. We heard from a minority of participants that they preferred to exercise alone and that having messages encouraging them to exercise with important others and answering survey questions about relatedness in exercise was somewhat unhelpful. Questions used to measure relatedness in PA and exercise have centered around exercising or being physically active with other people, often in group exercise contexts [66,84-86]. Thus, new approaches may be needed to support and measure relatedness in individually delivered PA interventions for people who prefer to exercise alone.

### Strengths and Limitations

This study has several strengths, including expanding a previously developed prototype intervention that used a psychometric approach to intervention development [75], using a systematic approach to develop and test the intervention (ie, the Obesity-Related Behavioral Intervention Trials and the National Institutes of Health’s Science of Behavior Change and Behavioral Intervention Development frameworks) and building off a solid base of observational and interventional research based on SDT and meaning. In addition, this technology-based intervention has a personalized text message delivery platform with built-in intelligence for smart SMS text messaging. This technology is easily scalable to large audiences. Moreover, in the pilot study, we had minimal missing data (33/35, 94% completed posttest assessments).

This study also has limitations. The study was designed as a proof-of-concept pilot trial. Participants served as their own controls instead of being compared to a no-intervention control group. The sample size was small, despite achieving adequate power to detect moderate within-person changes. These design choices were made to ensure that the intervention was related to changes in the hypothesized mechanisms; however, we are limited in our ability to state that the intervention was related to changes in PA or that increases in the hypothesized mechanisms caused changes in PA.

Finally, objectively measured PA using accelerometers had the advantage over self-report PA in reducing measurement bias [87]. However, it also required participants to wear the devices. Participants were encouraged to wear the devices on their nondominant wrist during daytime hours, and data were uploaded to a cloud-based data management system. However, our data suggest that the devices highly overestimated PA and did not provide accurate measurement of PA.

### Conclusions

MAP to Health is a resource-sparing, theory-based mHealth intervention that uses personalized EMIs to pair meaning with PA and support the SDT-based basic needs of autonomy, competence, and relatedness. This pilot study showed that this digital intervention was acceptable to participants and feasible to deliver, and it sufficiently engaged the target mechanisms of behavior change. Some modifications are needed to enhance the usability of the user interface, and future research is needed to test the efficacy of the intervention compared to a control group and examine whether the intervention effects on the hypothesized mechanisms of change account for changes in PA. If successful, this intervention could be widely disseminated, and the technological platform could be adjusted for interventions targeting a variety of behaviors and conditions.

## Abbreviations

CONSORT: Consolidated Standards of Reporting Trials
EMI: ecological momentary intervention
MAP: Meaningful Activity Program
MAS: Meaning Awareness Scale
mHealth: mobile health
MVPA: moderate to vigorous physical activity
PA: physical activity
PNES: Psychological Needs Satisfaction in Exercise Scale
REDCap: Research Electronic Data Capture
SDT: self-determination theory
SVS: Subjective Vitality Scale
